# Preparation of Butyl Acrylate Copolymer Emulsion and Its Regulation Effect on Cement Hydration

**DOI:** 10.3390/ma16072887

**Published:** 2023-04-05

**Authors:** Sifan Li, Zhongyang Mao, Min Deng

**Affiliations:** 1 College of Materials Science and Engineering, Nanjing Tech University, Nanjing 211800, China; 202061103061@njtech.edu.cn (S.L.); mzy@njtech.edu.cn (Z.M.); 2 State Key Laboratory of Materials-Oriented Chemical Engineering, Nanjing 211800, China

**Keywords:** cement hydration, butyl acrylate resin, temperature rise inhibitor

## Abstract

Due to its large volume and poor thermal conductivity, mass concrete is prone to temperature cracking caused by heat release during cement hydration after pouring. To address the issue of temperature cracking in mass concrete, this study utilized emulsion polymerization to prepare polybutyl acrylate (PBA) emulsions. At an optimal dosage of 1.5%, the addition of a PBA emulsion reduced the temperature rise of cement paste by 12.4%. The inhibitory mechanism of a PBA emulsion on cement hydration was analyzed by characterization techniques such as isothermal calorimetry, X-ray diffraction Rietveld full-profile fitting method (XRD), thermogravimetric–differential scanning calorimetry (TG-DSC), and mercury intrusion porosimetry (MIP). The results showed that the C_3_S content in the cement specimens with 1%, 1.5%, and 2% PBA increased by 13.83%, 23.52%, and 34.65% compared to the blank group, respectively, while the C_3_A content increased by 92.59%, 79.63%, and 96.30%, respectively. The addition of a PBA emulsion can slow down the hydration rate of C_3_S and C_3_A, thereby reducing the temperature rise and fall rate of cement hydration, reducing the peak heat release of the hydration reaction, and ultimately achieving the inhibition of the cement hydration reaction. In addition, the mechanical properties of PBA-modified cement-based materials were also tested. The results show that the addition of PBA can affect the early strength development of cement samples, but has no effect on the strength after 60 days. Therefore, PBA can be used as a hydration temperature rise control material to reduce the risk of temperature cracking in mass concrete.

## 1. Introduction

With the development of China’s construction industry, high-strength mass concrete has become one of the commonly used concretes in engineering [1]. However, for high-strength, large-volume concrete, which is commonly used in construction engineering, a significant challenge is posed by the large amount of heat generated by cement hydration after construction [2]. The reason is that, after the concrete is poured, a large amount of heat is released during cement hydration [3]. Sometimes, the hydration heat can cause the temperature of the concrete structure to rise to 70 or even 80 °C. During the process of gradually cooling down from the highest temperature to room temperature, the concrete undergoes thermal contraction. The restrained structural concrete may experience significant tensile stress, which eventually leads to cracking and failure of the concrete, affecting its durability and service life [4]. Therefore, the control of hydration heat should be considered in the preparation and design of high-strength, large-volume concrete. Based on the fact that the cause of temperature cracking in concrete is the heat released during cement hydration, taking measures to reduce the rate of cement hydration heat generation may potentially reduce the temperature rise of concrete in engineering structures and decrease the risk of cracking caused by thermal shrinkage during cooling. Chemical admixtures can significantly influence the heat of hydration of cement at very low dosages [5], which can control the hydration of cement from the source without changing the amount of cementitious material, reduce the temperature gradient of structural concrete, and lower the risk of temperature-related shrinkage cracking.

Polymers, as a type of functional chemical admixture, have wide applications in cementitious materials. They are commonly used in engineering repair and surface waterproof coatings. Commonly used water-based polymer dispersions include styrene–butadiene rubber (SBR) [6], styrene–acrylate emulsion (SAE) [7], and epoxy emulsion (EE) [8]. Many previous studies have shown that the impact of polymer emulsions on cement hydration is mainly related to factors such as their surface charge, functional groups in their molecular structure, and charge density. The retarding effect of polymers on cement hydration is consistent with the magnitude of their charge density, with higher charge density polymers having stronger retarding effects on cement hydration. In addition, polymers with anionic or cationic surface charges have a stronger adsorption capacity on cement surfaces than nonionic emulsions, thus having stronger inhibitory effects on cement hydration [9]. Different polymers have different functional groups that form different coordination bonds with hydration products, and different functional groups have different effects on hydration reactions. Polymers with ester groups in their molecular structure have stronger temperature control effects on cement-based materials than those with pure hydroxyl or carboxyl groups. This is because ester-based polymers undergo hydrolysis during the temperature rise of cement hydration, and the ester groups in the molecular formula can continuously hydrolyze to produce carboxyl groups, which can continuously inhibit cement hydration and achieve temperature control [10]. Currently, the research on polymer emulsions as a temperature rise inhibitor in water is mostly focused on their effect on the heat of hydration of cement, but the underlying mechanisms have not been thoroughly investigated. Further research is needed in our study to use more approaches to elucidate the influence mechanisms of polymer emulsion on cement hydration.

Of course, there are many substances in chemical admixtures that have temperature control properties for cement hydration, mainly including starch dextrin admixtures [11], hydroxy carboxylate salts [12], phase change materials [13], etc. However, most of these substances are powder chemical admixtures, and their dosage in cement is extremely low, so there are certain dispersion problems in their applications, resulting in unnecessary production costs. More importantly, the preparation of polymer emulsions through emulsion polymerization has the advantages of water dispersibility, which solves the current problem of the poor dispersibility of additives. The molecular structure of the polymer can be designed to synthesize different types of polymer emulsions, which can be used to explore the inhibition mechanism of cement hydration and have great potential in suppressing the temperature rise during the hydration of a large volume of cement; this is also a significant cause for choosing polymer emulsions as the research object in this paper. In this article, a polyester polymer was synthesized using the pre-emulsified, semi-continuous seed emulsion polymerization method, and its effects on cement hydration were investigated using adiabatic temperature rise testing, hydration heat, XRDrecelt quantitative analysis, TGA, and mercury porosimetry. The goal is to provide theoretical support for the synthesis of hydration temperature control materials.

## 2. Materials and Methods

### 2.1. Materials

#### 2.1.1. Raw Materials for Synthesizing Polymer Emulsions

As one of the top 10 non-cross-linking emulsions, styrene–acrylic emulsion possesses a strong molecular design ability due to the presence of unsaturated bonds in its molecular structure. It also has the advantages of simple synthesis process and high cost-effectiveness, which makes it a suitable research object for this experiment. Styrene (St, 99%) and butyl acrylate (BA, 99%) were monomers. Sodium dodecyl sulfate (SDS, 99%) was surfactant. Sodium persulfate (SPS, 99%) was initiator. All materials were provided by Shanghai National Pharmaceutical Chemicals Co., Ltd. (Shanghai, China), and without further purification.

#### 2.1.2. Cementitious Materials

**Figure 1:**
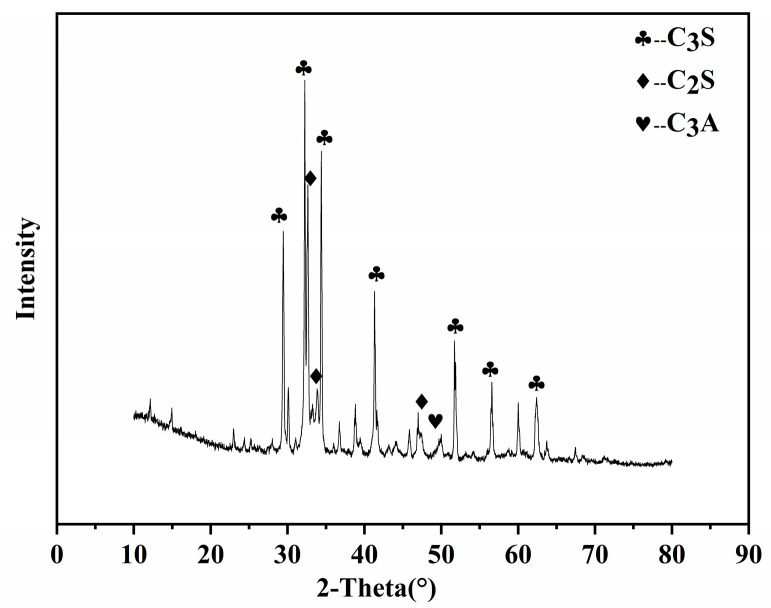
XRD (X-ray diffraction) pattern of cement.

The cement in the experiment was P·II 52.5 cement from Jiangnan-Xiaoyeda Cement Co., Ltd. (NanJing, China). The chemical compositions of the cement are shown in Table 1. The XRD pattern of the cement is shown in Figure 1.

#### 2.1.3. ISO Standard Sand

The standard sand in the experiment was provided by Xiamen Aisuo Standard Sand Co., Ltd. (Xiamen, China). The measured density of the sand is 2660 kg/m^3^ and the bulk density is 1800 kg/m^3^.

### 2.2. Methods

#### 2.2.1. Preparation Method of Polymer Emulsion

In the preliminary stage of the experiment, the emulsion conversion rate was used as a measure, an orthogonal experimental design was used to determine the type of emulsifier in the synthesis process, as well as the parameters such as reaction temperature, reaction time, and stirring speed. However, this part of the content will not be elaborated on in this article. In this study, the polymerization method was emulsion polymerization emulsion, and the reaction monomer was mixed by styrene (St) and butyl acrylate (BA) in a certain proportion. Sodium dodecyl sulfate (SDS) was the emulsifier, and sodium persulfate (NDS) was the initiator. The polymerization was carried out by forming an emulsion in water under mechanical stirring. The specific steps for preparing the polymer emulsion using emulsion polymerization are as follows: First, the reactants are poured into a flask that is equipped with a stirrer in a certain proportion to form a pre-emulsion under high-speed stirring. Under nitrogen protection, the reaction temperature is raised to 75 °C, and 1/3 of the pre-emulsion is added to the three-necked flask. After 90 min of reaction, another 1/3 of the pre-emulsion is added to the reaction system while maintaining a temperature of 75 °C, and the reaction is continued for another 90 min. The remaining 1/3 of the pre-emulsion is added to the reaction system, and the reaction is continued for another 90 min at 75 °C. Finally, the reaction system is heated to 80 °C and matured for 60 min. After cooling to room temperature, the reaction is completed. The relevant physical parameters of the synthesized emulsion will be detailed in the following chapters. The specific composition of the polymerization reaction is shown in Table 2. The properties of the polymer emulsion meet the standard “Polymer Emulsion for Building Waterproof Materials” (JC/T 1017-2020) [14] and “Evaluation Method of Emulsifying Performance for Medium and High Viscosity Emulsions” (GB/T 11543-2008) [15].

#### 2.2.2. Characterization of Polymer Emulsion

The polymer emulsion needs to be purified before characterization to remove unreacted small molecule monomers and emulsifiers. Since the monomers and emulsifiers are soluble in ethanol while the polymer is insoluble, the polymer emulsion can be purified by washing with ethanol to remove the monomers and emulsifiers.

- Solid content test.
- The steps for determining the solid content of an emulsion are as follows:
- Prepare the sample: Dry a flat-bottomed weighing bottle with a diameter of 50 mm in a constant temperature drying oven until it reaches a constant weight. Weigh 1 g of the prepared polymer emulsion sample into the weighing bottle (accurate to 0.0001 g) and allow it to self-level. Place the bottle in the drying oven with the shelf positioned at a height of 2/3 of the oven, and control the temperature at around 105 °C. After drying to a constant weight, remove the bottle and cool it to room temperature in a desiccator before weighing.
- The solid content (S) is calculated using the following formula:
- $S=\frac{m2-m1}{m}\times 100\%$.
- M—the weight of the original sample.
- m1—the weight of the weighing bottle before adding the sample.
- m2—the weight of the weighing bottle with the dried sample.
- Centrifugal stability test.
- Inject the emulsion into a centrifuge tube, centrifuge at a speed of 4000 r·min^−1^ for 15 min in a centrifuge, and observe if it separates into layers. The polymer emulsion prepared in this study does not separate into layers within six months and exhibits good stability.
- Fourier-transform infrared (FTIR).
- FTIR (Fourier-transform infrared) spectroscopy was used to characterize the polymer emulsion. The instrument used was Nicolet IS5 FTIR from Thermo Fisher in Massachusetts, USA. The purified polymer was dissolved in ethyl acetate and dropped onto a potassium bromide (KBr) plate, and then dried to form a thin film. The sample was measured in transmission mode over the range of 4000–400 cm^−1^ with a resolution of 4 cm^−1^ and 32 scans. In the infrared spectrum, the x-axis represents the wavenumber and the y-axis represents the absorbance.
- Gel permeation chromatography (GPC).
- The synthesized polymer emulsion molecular weight and molecular weight distribution were determined using gel permeation chromatography (GPC) with triple detectors. The specific testing procedure is as follows: a mixture of a certain amount of sample with a concentration of 0.003 mg/mL and water was prepared and injected into the sample tube. The specific parameters of the GPC were set as follows: flow rate of 1 mL/min, minimum pressure of 0 MPa, maximum pressure of 10 MPa, flow slope of 1.2 mL/min^2^, and temperature of 20 °C.
- Measurement of particle size distribution.
- Mastersizer 2000 laser particle size distributor was used for the particle size distribution test, manufactured by Malvern Instruments LTD. The particle size of polymer emulsion was measured by laser particle size analyzer and its particle size distribution curve was drawn.

#### 2.2.3. Testing of Cement-Based Materials Properties

**Figure 2:**
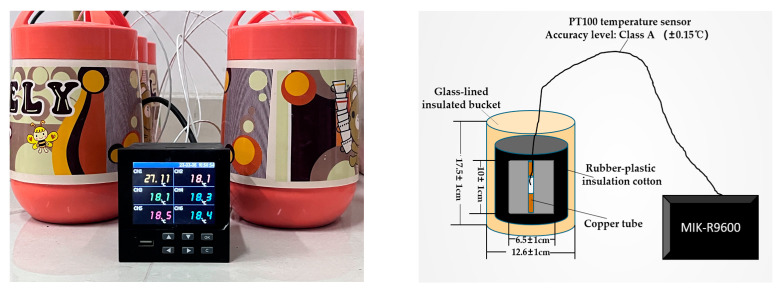
Semi-adiabatic calorimeter apparatus diagram and its cross-sectional view.

- Semi-adiabatic temperature rise.
- The semi-adiabatic temperature rise test was performed using a laboratory-made, semi-adiabatic temperature rise apparatus. As shown in Figure 2, which utilizes the insulation properties of a glass thermal insulation bucket and rubber insulation to effectively reduce the convective heat loss from the surface of concrete specimens. This allows smaller laboratory specimens to achieve a temperature rise similar to that of large-volume concrete in actual engineering, and can be used to evaluate the temperature control effect of hydration control materials. The cement-based material is added in the form of emulsion; W/C is 0.4, and the water is composed of the water contained in the example together with additional tap water.
- Macroscopic performance.
- According to Chinese Standard “Testing Method for Cement Mortar Strength” (GB/T 17671-2021) [16], steel molds of 40 mm × 40 mm × 160 mm are used for molding. Before molding, the steel plates of the mold are sealed with plastic wrap to prevent the MKPC from sticking to the mold and making it difficult to demold. After the mortar is molded, the mold is demolded within 24 h and cured at a specified age in air with a temperature of 20 ± 3 °C and a relative humidity of 50%. Flexural strength and compressive strength are tested on an AEC-201 cement strength testing machine.

#### 2.2.4. Hydration Kinetics Characterization

- Isothermal calorimetry.
- The hydration heat differences of cement pastes at different ages were measured using TAM Air eight-channel calorimeter produced by Thermometric AB in Sweden. The testing temperature was 20 °C and the water–cement ratio was 0.4.
- XRD.
- X-ray diffraction analysis (XRD): X-ray diffraction analysis was performed using a SMART LAB X-ray diffractometer produced by RIGAKU Corporation of Japan. The powder sample diffraction method was used, and the operating conditions of the instrument were controlled to use Cu target K radiation, tube voltage of 40 kV, and tube current of 100 mA. The sample was dried at 60 °C, ground to a fine powder, and passed through an 80 μm sieve using an agate mortar. The sample was filled into a glass sample holder with a depth of 0.5 mm, and the surface of the powder was lightly pressed with a smooth glass surface before measurement using an X-ray diffractometer. The scan speed was set to 10°/min, and the scanning angle range was 10° to 70°.
- TG-DSC.
- The present study used a STA-449C simultaneous thermal analyzer produced by Netzsch GmbH (Germany) to conduct experiments on cementitious materials. Cement paste specimens cured to the specified age were cut into small pieces, soaked in ethanol for 7 days to terminate cement hydration, and then dried at 60 °C. These small pieces were ground into powder to obtain the testing samples. In the experiments, 10 mg to 15 mg of samples were taken, the temperature range was set between 20 °C and 1000 °C, the linear heating rate was 10 °C/min, and nitrogen gas was used as the environment (20 mL/min nitrogen flow). The CH content in the samples was determined by the thermal weight loss in the temperature range of 400–550 °C of the CH decomposition endothermic peak determined by DSC, and normalized to the mass percentage of the original cementitious components in the sample.
- MIP.
- The cement paste was taken out at a specified age of curing and immersed in anhydrous ethanol solution for 72 h to terminate the cement hydration. After drying in an oven, the broken samples were selected and particles with a diameter of 3–5 mm were chosen for mercury intrusion porosimetry (MIP) experiments. The Poremaster GT-60 instrument was used to analyze the pore structure of the cement paste through mercury intrusion testing. By collecting the amount of mercury intrusion at different pressures, the volume corresponding to different pore sizes can be obtained, and the pore size distribution can be calculated, thereby analyzing the effect of polymer emulsion on the pore structure of cement paste from a microscopic perspective.

## 3. Results and Discussion

### 3.1. Basic Information of Polymer Emulsion

The physical information of polymer emulsions conforming to the JC/T 1017-2020 [14] and GB/T 11543-2008 [15] standards is shown in Table 3.

**Figure 3:**
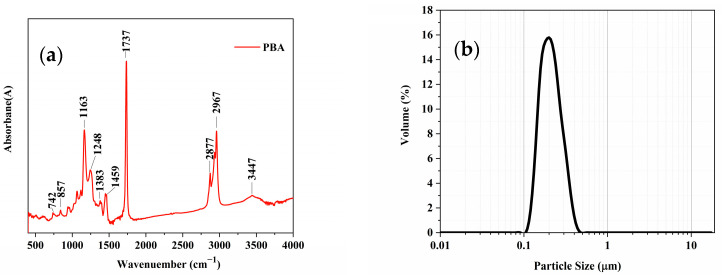
Characterization of polymer emulsion. (**a**) Infrared spectrogram. (**b**) Grain size distribution map.

The infrared spectrum of the polymer emulsion is shown in Figure 3a. The absorption peaks at 1248 cm^−1^ and 1163 cm^−1^ correspond to the asymmetric stretching vibration of C-O-C in the ester group. The peak at 1737 cm^−1^ is attributed to the asymmetric stretching vibration of C=O, and its overtone peak is observed at 3447 cm^−1^. The absorption peaks at 2967 cm^−1^, 2877 cm^−1^, and 1383 cm^−1^ are assigned to the anti-symmetric stretching vibration, symmetric stretching vibration, and out-of-plane bending vibration of C-H in methyl and methylene groups, respectively. The peak at 1459 cm^−1^ corresponds to the plane bending vibration of O-H in -COOH. The absorption peaks at 742 cm^−1^ and 857 cm^−1^ are attributed to the single substitution absorption peaks of the benzene ring. The absorption peaks in the range of 3085–3025 cm^−1^ are assigned to the stretching vibration of C-H attached to C=C, and those in the range of 1680–1600 cm^−1^ correspond to the stretching vibration of C=C. These results indicate that there is no double bond in the polymer, and both styrene and butyl acrylate participate in free-radical polymerization.

### 3.2. Semi-Adiabatic Temperature Rise

**Figure 4:**
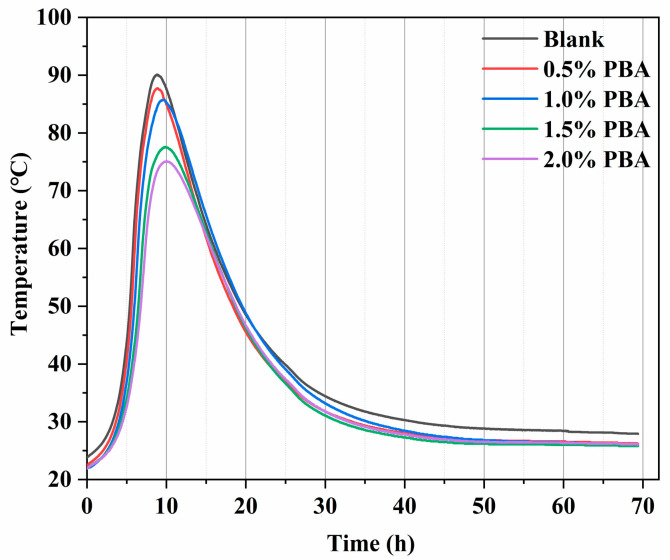
The effect of PBA on the hydration temperature rise of cement.

Figure 4 shows the effect of polybutyl acrylate emulsion (PBA) on the temperature rise during cement hydration. According to the figure, with the increase in the PBA dosage, the peak temperature of cement hydration in the slurry is significantly reduced. Compared with the highest hydration temperature of 90.0 °C in the blank group, PBA reduces it by 2.2 °C at a dosage of 0.5%; at a dosage of 1%, the highest temperature is reduced by 4.4 °C; at a dosage of 1.5%, the highest temperature is reduced by 12.4%, a decrease of 13.8%; and at a dosage of 2%, the highest temperature is reduced by 14.9 °C (16.6%). Compared with the cooling effect of the styrene–butadiene latex, PBA can achieve a good cooling effect at a lower dosage. The addition of PBA has little effect on the time of the hydration temperature peak in cement. In the cases of PBA dosages of 0.5%, 1%, 1.5%, and 2%, the appearance time of the hydration temperature peak in cement is delayed by 0 h, 0.8 °C, 1.0 °C, and 1.1 °C, respectively. Therefore, the cooling effect of PBA is significant, with little influence on the time of the temperature peak, and it can slow down the heating and cooling rate of the hydration temperature rise.

### 3.3. Characterization of Cement Hydration Kinetics

#### 3.3.1. Isothermal Calorimetry

**Figure 5:**
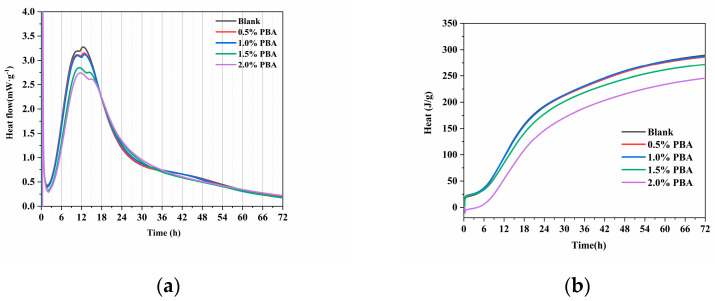
Effect of sugars on the heat flow curve of cement hydration. (**a**) Hydration heat rate. (**b**) Cumulative heat release curve.

According to the heat flow curve of the cement hydration process, the heat flow curve of the cement can be divided into the following typical stages: the initial dissolution stage (I), the induction period (II), the acceleration period (III), and the declining period (IV) [17]. The influence of different PBA dosages on the heat evolution process of cement hydration is shown in Figure 5. Figure 5a shows the heat release curve, while Figure 5b shows the cumulative heat release. From the figure, it can be seen that the heat release curve of cement hydration has two hydration peaks. The first peak is caused by the hydration of aluminate to form AFt, and the second peak is caused by the formation of C-S-H and CH from the hydration of C_3_S. Since the heat release of cement hydration is mainly concentrated in the second heat release peak, the focus is on the effect of PBA on the second heat release peak. Compared with the blank, the temperature rise and fall rates of cement hydration are both slowed down with the increase in the PBA dosage, and the degree of reduction in hydration heat peak is basically positively correlated with the increase in the PBA dosage. The hydration heat rate peak value of a 2% PBA dosage in cement hydration is 2.74 mW/g, which is 16.5% lower than the peak value of 3.28 mW/g for the blank. The addition of a polymer emulsion significantly inhibits cement hydration, and this inhibitory effect gradually increases with the increase in the emulsion dosage. The reason for this may be that the polymer enriches calcium ions around itself through electrostatic or chelation effects, which further causes the enrichment of silicate ions around it. On one hand, the adsorption of the emulsion on the surface of cementitious silicate minerals leads to the enrichment of the calcium ion concentration, which reduces the surface dissolution rate of cementitious silicate minerals [18]. On the other hand, ions in the liquid phase form ion-enriched regions around the emulsion particles on the surface of cement particles, which further disperses the limited concentration of silicate anions on the surface of cement particles and delays the nucleation rate of C-S-H crystals [19]. Therefore, the inhibition effect of emulsion on cement hydration is strong.

The selected main parameters of the cement hydration heat curve are listed in Table 4. Ratemax represents the maximum heat release rate, and Q_24 h_, Q_48 h_, and Q_72 h_ represent the cumulative heat release at hydration ages of 24 h, 48 h, and 72 h (3 days), respectively. When the PBA content is below 1%, it has little effect on the heat release rate and the total heat release of cement hydration. When the PBA content exceeds 1%, with the increase in the PBA content, the cumulative heat release of cement hydration at each age is lower than that of the blank group. The 24 h heat release of the sample with 2% PBA content is 145.10 J, which is 24.1% lower than that of the blank sample, which is 191.28 J. With the extension of time, there is still a difference in heat release between the sample with 2% PBA content and the blank group at 72 h, and more research is needed on the mechanism of PBA’s influence on cement hydration.

#### 3.3.2. XRD

Quantitative analysis of phase composition in cementitious materials using an X-ray diffraction with Rietveld refinement. This method is a quantitative analysis method based on X-ray diffraction, which can determine the content and structural parameters of phases in a sample by fitting experimental diffraction data with model diffraction data. In the hydration process of cement paste, the main hydration products generated include calcium silicate hydrate (C-S-H), calcium hydroxide (CH), and hydrated calcium aluminate (AFt) [20]. The crystal structure and relative content of these hydration products can be determined through an XRD Rietveld analysis [21]. It should be noted that the XRD Rietveld method can only quantitatively analyze the crystalline phases in cement paste, and its analysis of amorphous phases is limited. Therefore, it is necessary to combine other analytical methods such as thermal analysis and infrared spectroscopy to comprehensively analyze the composition of hydration products in cement paste.

**Figure 6:**
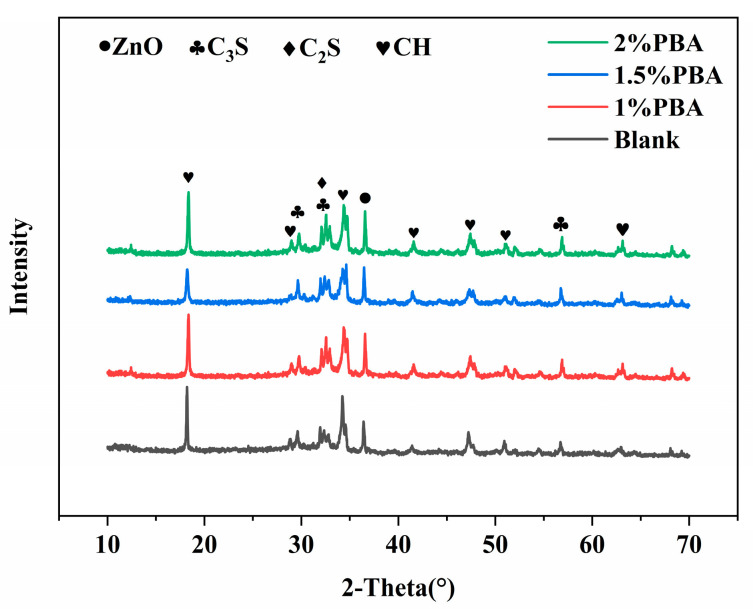
The effect of PBA on the early phase composition of cement hydration.

As shown in Figure 6, the addition of PBA affects the content of the hydration reactants, with the C_3_S and C_3_A content in PBA cement samples higher than those in the blank group. The C_3_S content in PBA cement samples with a 1%, 1.5%, and 2% dosage of PBA increased by 13.83%, 23.52%, and 34.65%, respectively, compared to the blank group. The C_3_A content increased by 92.59%, 79.63%, and 96.30%, respectively, indicating that PBA has a greater effect on the hydration of C_3_A than C_3_S. At the same time, the addition of PBA hinders the formation of the hydration product CH, with the amount of CH generated decreasing by 20.77%, 20.59%, and 28.19% with an increasing PBA emulsion dosage. Overall, the effect of a PBA emulsion on cement hydration is mainly achieved by suppressing the early hydration of C_3_S and C_3_A, and its inhibitory effect on the hydration of C_3_A is much greater than that on C_3_S (Table 5). This mainly slows down the hydration reaction rate of C_3_S in cement, thereby reducing the heat release rate and achieving the effect of temperature control.

#### 3.3.3. TG-DSC

**Figure 7:**
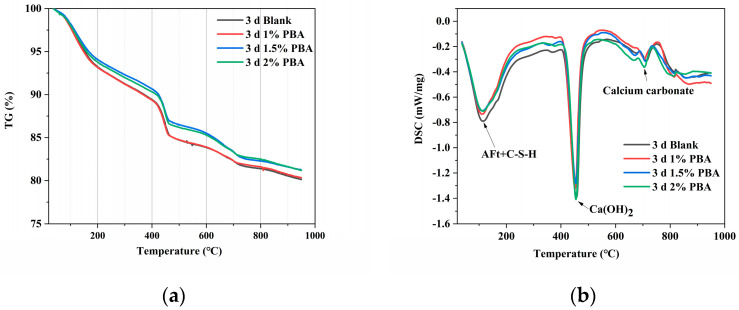
TG-DSC curves of cement slurries with different PBA contents at three days. (**a**) TG curve and (**b**) DSC curve.

Thermogravimetric (TG) curves of cement samples with different PBA contents in 3 days of hydration are shown in Figure 7. It can be seen that each sample’s TG curve exhibits three weight losses, and the three weight losses correspond to the three endothermic peaks in the DSC curve. According to the literature, it is known that the mass loss below 350 °C is considered to be the result of the dehydration of calcium silicate hydrates, calcium aluminate hydrates, and calcium sulfoaluminate hydrates during cement hydration; the mass loss between 400 and 550 °C is related to the decomposition of Ca(OH)_2_ (CH); and the mass loss in the temperature range of 650–750 °C is due to the decomposition of CaCO_3_ [21].

To quantitatively analyze the content of Ca(OH)_2_ using TG-DSC, the TG curves of different cement paste samples are converted into differential thermogravimetric (DTG) curves using Origin 2018 in order to determine the starting and ending temperatures of the decomposition reaction of CH [22]. Then, based on the TG curve, the weight loss rate of the cement paste can be determined, which allows for the quantitative analysis of the content of CH. Based on the analysis of TG-DSC data, it was found that the mass loss rates of the cement samples with a PBA emulsion at 0%, 1%, 1.5%, and 2% during the 3-day hydration process were 4.58%, 4.53%, 4.46%, and 4.15%, respectively, in the temperature range of 400–550 °C. With the increase in PBA emulsion dosage, the mass loss rate of the cement sample in the range of 400–550 °C gradually decreased, indicating that the CH content in the cement paste with PBA emulsion was relatively low and the content of CH was reduced. The addition of the PBA emulsion hindered the generation of CH and thereby suppressed the hydration process of the cement.

Using the same method, the increase in the content of the carbonate phase was also calculated, and the loss curve of calcium carbonate at 700 °C also indicated an increase in the content of the carbonate phase. This is because the hydrolysis of the polymer emulsion produces carboxyl groups, which react with Ca^2+^ in the slurry to form calcium formate or calcium acetate, which ultimately decompose at high temperatures to generate calcium carbonate [20], as shown in Equation (1):

(1) $$\mathrm{Ca}{\left({\mathrm{CH}}_{3}\mathrm{COO}\right)}_{2}\overset{380-400{}^{\circ}C}{\to }{\mathrm{CH}}_{3}{\mathrm{COCH}}_{3}+{\mathrm{CaCO}}_{3}$$

#### 3.3.4. MIP

**Figure 8:**
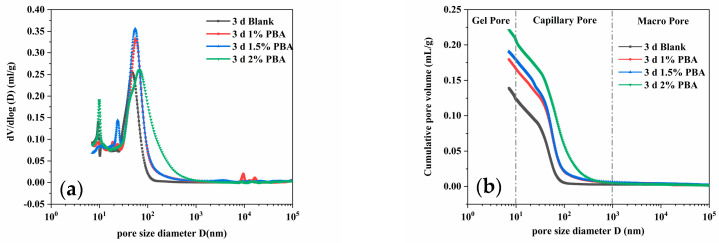
Pore structure of samples with different PBA content for 3 days: (**a**) pore size distribution and (**b**) cumulative porosity.

**Figure 9:**
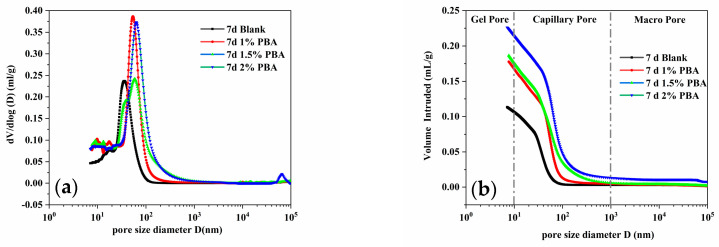
Pore structure of samples with different PBA content for 7 days: (**a**) pore size distribution and (**b**) cumulative porosity.

Figure 8 and Figure 9 show the test results of the pore size distribution integral curves and differential curves of cementitious materials with different admixture dosages at different ages. The pore size distribution and structure of cementitious materials can be quantitatively described by obtaining a curve of the volume of each pore size and the corresponding pressure under different pressure levels through MIP testing, which is represented by the integral curve and differential curve of the pore size distribution [23]. Based on the size of the internal pores in the cement specimen, they can be divided into gel pores, capillary pores, and air pores, with diameters smaller than 10 nm, 10–1000 nm, and greater than 1000 nm, respectively. The cumulative pore size distribution curves show that the pore structure of the cement paste exhibits a typical unimodal distribution [24]. The addition of PBA expands the pore size distribution range of the cement samples and increases the percentage of pores within this range, regardless of the PBA content. This is one of the reasons for the decrease in the mechanical properties of the cement specimens. Furthermore, with an increasing dosage of the PBA emulsion, the total porosity of the cement samples increased by 23.23% (1%), 16.47% (1.5%), and 28.19% (2%) after 3 days, respectively (Table 6). After 7 days of hydration, the total porosity of the cement samples increased from 20.12% to 29.29%, 27.20%, and 35.58%, respectively (Table 7). This may be because when polymers are added to a cement slurry, their molecular weight is larger and the intermolecular interaction forces are stronger, causing the polymers to interact with the particles in the cement, and forming certain aggregates [25]. These aggregates may be poorly dispersed in the cement, leading to a weaker connection between cement particles and the formation of more pores and defects, resulting in an increase in the porosity of the cement slurry. In addition, the presence of polymers in the cement slurry can also affect the progress of the cement hydration reaction, which may affect the surface activity of cement particles and make the surface structure of cement particles irregular, leading to an increase in the porosity of the cement slurry.

### 3.4. Compressive and Flexural Strength

**Figure 10:**
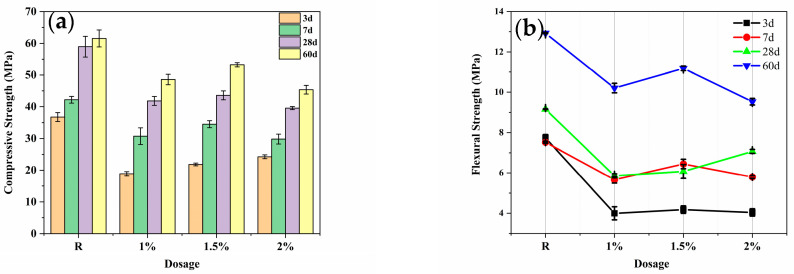
Compressive and flexural strength of cement mortars with addition of the different polymers at different ages. (**a**) Compressive strength and (**b**) Flexural strength.

Under standard curing conditions, the effect of different amounts of PBA emulsions on the compressive and flexural strength of cement specimens is shown in Figure 10. As can be seen from Figure 10a, the compressive strength of the cement specimens with an added PBA emulsion was lower than that of the blank group. Compared with the blank group, the compressive strength of the cement specimens with a PBA emulsion at 0%, 1%, 1.5%, and 2% decreased by 29.09%, 26.10%, and 32.94%, respectively, at 28 days. This indicates that the addition of PBA emulsions has a significant impact on the compressive strength of cementitious materials. Moreover, at 60 days, the strength of the cement specimen with a 1.5% addition reached 53.25 MPa. The main reason for the effect of polymer emulsion on the compressive strength of cement specimens is that PBA significantly delays the hydration of cement and reduces the total heat of hydration, thereby exacerbating the degradation of the compressive strength of cement. In addition, there is a linear relationship between the compressive strength and the porosity of cement slurry [26]; the higher the porosity, the lower the cement strength, and the lower the porosity, the higher the cement strength. With the addition of PBA, the total porosity of the cement slurry shows a trend of first increasing and then decreasing. Therefore, at a dosage of 1.5%, the total porosity increases less when compared to the blank group, resulting in a relatively small impact on the strength. Figure 10b shows the effect of the same dosage of PBA emulsion on the flexural strength of cement specimens. With the increase in the PBA emulsion dosage, its effect on the flexural strength of cement specimens first decreases and then increases, and there exists an optimal dosage [27]. At the optimal dosage of 1.5%, the flexural strength at 60 d reaches 13.07 MPa, which may be attributed to the formation of a network structure of a polymer emulsion inside the cement slurry to improve its density. Therefore, the influence of PBA emulsions on the compressive and flexural strength of cement slurry is attributed to two aspects: delaying the hydration reaction of cement and reducing the total heat release. Due to the delayed hydration reaction caused by the reaction between PBA and cement, the total heat release is reduced, leading to a decrease in the compressive and flexural strength of the cement paste. Additionally, the addition of a PBA emulsion can create certain pore structures, resulting in an increase in the internal pore volume of the cement paste, which also affects its compressive and flexural strength. Furthermore, the addition of a PBA emulsion may form a certain network structure within the cement paste, enhancing its compactness and affecting its flexural strength [28].

In summary, the addition of a PBA emulsion can have varying degrees of impact on the compressive and flexural strength of cementitious materials, and its influencing factors include the dosage of PBA and the curing time of the cementitious materials. Under certain conditions, the addition of a PBA emulsion may have a positive effect on optimizing the performance of cementitious materials, but the appropriate dosage range and potential side effects should also be considered.

## 4. Conclusions

In this study, polybutyl acrylate (PBA) latex was synthesized by emulsion polymerization, which exhibited excellent stability and no layering after long-term storage. The inhibitory mechanism of PBA on the hydration of cement was investigated by an isothermal calorimetry, XRD quantitative analysis, thermogravimetric analysis, and mercury intrusion porosimetry. The analysis results lead to the following conclusions:

1. The results of the semi-adiabatic temperature rise experiment show that the addition of PBA can effectively control the temperature rise during cement hydration. When the dosage of PBA is 1.5%, the highest temperature rise during cement hydration can be reduced by 12.4 °C. Based on the isothermal calorimetry results, it can be concluded that the effect of PBA on the heat evolution of cement hydration is mainly reflected in slowing down the heating and cooling rates, reducing the hydration peak, and the retarding and cooling effects become stronger with the increase in the PBA content. Through the analysis of the results, the butyl acrylate copolymer emulsion prepared in this paper not only solves the dispersibility problem of the conventional starch-based hydration temperature rise inhibitor in the process of use [5], but also, compared with other emulsion admixtures [19], it can control the hydration temperature rise of cement better at a low dosage.
2. Characterized by XRD and TGA, and revealing the inhibitory mechanism of PBA on cement hydration. The mass loss rates of the cement samples with a PBA emulsion at 0%, 1%, 1.5%, and 2% during the 3-day hydration process were 4.58%, 4.53%, 4.46%, and 4.15%, respectively. This demonstrates that the addition of PBA not only suppresses the early hydration of C_3_S and C_3_A, but also has a stronger inhibitory effect on the hydration of C_3_A. Additionally, it hinders the formation of hydration products such as CH and CaCO_3_.
3. The addition of PBA widened the pore size distribution range of the cement specimen and increased the pore size percentage within this range. With an increasing dosage of the PBA emulsion, the total porosity of the cement samples increased by 23.23% (1%), 16.47% (1.5%), and 28.19% (2%) after 3 days, respectively. This may be due to the large molecular weight of the polymer, which leads to poor dispersion in the cement and weakens the connections between cement particles, resulting in more pores and defects. This is also one of the reasons for the decrease in the mechanical properties of the cement specimens. At a PBA content of 1.5%, the 60-day strength of the cement specimen reached the standard strength range. There is an optimal dosage range for PBA emulsion application, and the appropriate dosage range and potential side effects should be considered when using it.

## Figures and Tables

**Table 1:** Chemical composition of cementitious materials (wt%).

Material	CaO	SiO_2_	Al_2_O_3_	Fe_2_O_3_	SO_3_	MgO	K_2_O	LOI
cement/%	63.66	20.87	4.42	2.89	2.10	0.94	0.51	3.30

**Table 2:** The reaction components and their proportions for emulsion polymerization.

Name	Dispersion Phase	Organic Phase	Emulgator	Catalyzer
Proportion (%)	65.2	32.6	2.0	0.2

**Table 3:** Basic physical information of polymer emulsions.

Sample	Appearance	pH	Solid Content (%)	Stability	Particle Size (nm)	Mw (g/mol)
PBA	Milky white with bluish	11	50	No delamination	203	46,700

**Table 4:** Changes in the maximum hydration rate and heat of hydration of cement pastes with different dosages of PBA.

Sample	Rate_max_ (mW g^−1^)	Q_24 h_ (J g^−1^)	Q_48 h_ (J g^−1^)	Q_72 h_ (J g^−1^)
Blank	3.28	191.28	257.66	290.11
0.5% PBA	3.15	190.44	256.61	285.72
1% PBA	3.12	191.06	258.81	287.71
1.5% PBA	2.85	176.41	243.84	271.48
2% PBA	2.74	145.10	215.57	245.41

**Table 5:** Composition (wt%) of cement hydration slurry by Rietveld refinement.

Sample	C_3_S	C_2_S	C_3_A	C_4_AF	CH
Blank	11.86	12.31	0.54	6.40	16.85
1% PBA	13.50	9.89	1.04	6.27	13.35
1.5% PBA	14.65	13.20	0.97	8.13	13.38
2% PBA	15.97	13.60	1.06	6.94	12.81

**Table 6:** Total porosity of cement sample at 3 days.

Sample	3 d Blank	3 d 1.0% PBA	3 d 1.5% PBA	3 d 2.0% PBA
Porosity Summary (%)	23.68	29.18	27.58	33.99

**Table 7:** Total porosity of cement sample at 7 days.

Sample	7 d Blank	7 d 1.0% PBA	7 d 1.5% PBA	7 d 2.0% PBA
Porosity Summary (%)	20.12	29.29	27.20	34.58

## Data Availability

The data presented in this study are available on request from the corresponding author.

## References

[1] Xin J., Jiang X., Chen Z., Zuo L., Zhang G., Wang Z., Qi C., Zhang L., Liu Y. (2022). Early Age Thermal Cracking Resistance of Basalt Fiber-Reinforced Concrete for Mass Concrete Structures under Restraint Condition. Structures.

[2] Klemczak B., Batog M., Pilch M., Żmij A. (2017). Analysis of Cracking Risk in Early Age Mass Concrete with Different Aggregate Types. Procedia Eng..

[3] Chiniforush A.A., Gharehchaei M., Nezhad A.A., Castel A., Moghaddam F., Keyte L., Hocking D., Foster S. (2022). Numerical Simulation of Risk Mitigation Strategies for Early-Age Thermal Cracking and Def in Concrete. Constr. Build. Mater..

[4] Khalel H.H.Z., Khan M., Starr A. (2023). Dynamic Response-Based Crack Resistance Analysis of Fibre Reinforced Concrete Specimens under Different Temperatures and Crack Depths. J. Build. Eng..

[5] Yan Y., Wang R., Wang W., Yu C., Liu J. (2021). Effect of Starch-Based Admixtures on the Exothermic Process of Cement Hydration. Constr. Build. Mater..

[6] Deredas K., Kępczak N., Urbaniak M. (2021). Influence of Doping with Styrene-Butadiene Rubber on Dynamic and Mechanical Properties of Polymer Concrete. Compos. Struct..

[7] Bahranifard Z., Vosoughi A., Tabrizi F.F., Shariati K. (2022). Effects of Water-Cement Ratio and Superplasticizer Dosage on Mechanical and Microstructure Formation of Styrene-Butyl Acrylate Copolymer Concrete. Constr. Build. Mater..

[8] Pang B., Jin Z., Zhang Y., Xu L., Li M., Wang C., Zhang Y., Yang Y., Zhao P., Bi J. (2022). Ultraductile Waterborne Epoxy-Concrete Composite Repair Material: Epoxy-Fiber Synergistic Effect on Flexural and Tensile Performance. Cem. Concr. Compos..

[9] Yuan Q., Xie Z., Yao H., Huang T., Fan M. (2022). Hydration, Mechanical Properties, and Microstructural Characteristics of Cement Pastes with Different Ionic Polyacrylamides: A Comparative Study. J. Build. Eng..

[10] Lu Z., Kong X., Zhang Q., Cai Y., Zhang Y., Wang Z., Dong B., Xing F. (2016). Influences of Styrene-Acrylate Latexes on Cement Hydration in Oil Well Cement System at Different Temperatures. Colloids Surf. A Physicochem. Eng. Asp..

[11] Yan Y., Scrivener K.L., Yu C., Ouzia A., Liu J. (2021). Effect of a novel starch-based temperature rise inhibitor on cement hydration and microstructure development: The second peak study. Cem. Concr. Res..

[12] Pourchez J., Govin A., Grosseau P., Guyonnet R., Guilhot B., Ruot B. (2006). Alkaline stability of cellulose ethers and impact of their degradation products on cement hydration. Cem. Concr. Res..

[13] Feng Q., Liu X.J., Peng Z.G., Zheng Y., Huo J.H., Liu H. (2019). Preparation of low hydration heat cement slurry with micro-encapsulated thermal control material. Energy.

[14] (2021). Polymer Emulsions for Building Waterproofing Materials.

[15] (2008). Surface Active Agents—The Identification of Emulsion for Moderate Tohigh Viscosity and the Evaluation Method of Emulsifying Capability.

[16] (2021). Test Method of Cement Mortar Strength(ISO Method).

[17] Jansen D., Goetz-Neunhoeffer F., Lothenbach B., Neubauer J. (2012). The Early Hydration of Ordinary Portland Cement (Opc): An Approach Comparing Measured Heat Flow with Calculated Heat Flow from Qxrd. Cem. Concr. Res..

[18] Huo J., Wang Z., Zhang T., He R., Chen H. (2021). Influences of Interaction Between Cement and Ionic Paraffin Emulsion on Cement Hydration. Constr. Build. Mater..

[19] Kong X., Emmerling S., Pakusch J., Rueckel M., Nieberle J. (2015). Retardation Effect of Styrene-Acrylate Copolymer Latexes on Cement Hydration. Cem. Concr. Res..

[20] Gonçalves B., Exposito C., Ishikawa T.T., Koga G.Y. (2023). X-Ray Diffraction Study of the Early Hydration of Portland Cements Containing Calcium Carbonate by in-Situ and Ex-Situ Approaches. Constr. Build. Mater..

[21] De Oliveira A.M., Oliveira A.P., Vieira J.D., Junior A.N., Cascudo O. (2023). Study of the Development of Hydration of Ternary Cement Pastes Using X-Ray Computed Microtomography, Xrd-Rietveld Method, Tg/Dtg, Dsc, Calorimetry and Ftir Techniques. J. Build. Eng..

[22] Cai J., Zhang C., Zeng L., Xu H., Wang J., Liu K., Cheng X. (2021). Preparation and Action Mechanism of Temperature Control Materials for Low-Temperature Cement. Constr. Build. Mater..

[23] Xue S., Meng F., Zhang P., Bao J., Wang J., Zhao K. (2020). Influence of Water Re-Curing on Microstructure of Heat-Damaged Cement Mortar Characterized by Low-Field Nmr and Mip. Constr. Build. Mater..

[24] Silva D.A., John V.M., Ribeiro J., Roman H.R. (2001). Pore Size Distribution of Hydrated Cement Pastes Modified with Polymers. Cem. Concr. Res..

[25] Zhang X., Du M., Fang H., Shi M., Zhang C., Wang F. (2021). Polymer-Modified Cement Mortars: Their Enhanced Properties, Applications, Prospects, and Challenges. Constr. Build. Mater..

[26] Ramli M., Tabassi A.A. (2012). Effects of polymer modification on the permeability of cement mortars under different curing conditions: A correlational study that includes pore distributions, water absorption and compressive strength. Constr. Build. Mater..

[27] Shi X., Cheng J., Xu L., Feng T., Han J., Zhang P., Guo Z. (2022). Study on the Effect of Wer and Eva on the Performance and Microstructure of Cement Mortars for a Prefabricated Residential Floor. J. Build. Eng..

[28] Aghaee K., Sposito R., Thienel K., Khayat K.H. (2023). Effect of Additional Water or Superplasticizer on Key Characteristics of Cement Paste Made with Superabsorbent Polymer and Other Shrinkage Mitigating Materials. Cem. Concr. Compos..

